# Hyponatraemia in Emergency Medical Admissions—Outcomes and Costs

**DOI:** 10.3390/jcm3041220

**Published:** 2014-10-29

**Authors:** Richard Conway, Declan Byrne, Deirdre O’Riordan, Bernard Silke

**Affiliations:** Department of Internal Medicine, St James’s Hospital, Dublin 8, Ireland; E-Mails: drrichardconway@gmail.com (R.C.); declangbyrne@me.com (D.B.); doriordan@stjames.ie (D.R.)

**Keywords:** hyponatraemia, mortality, length of stay, emergency admission

## Abstract

Healthcare systems in the developed world are struggling with the demand of emergency room presentations; the study of the factors driving such demand is of fundamental importance. From a database of all emergency medical admissions (66,933 episodes in 36,271 patients) to St James’ Hospital, Dublin, Ireland, over 12 years (2002 to 2013) we have explored the impact of hyponatraemia on outcomes (30 days in-hospital mortality, length of stay (LOS) and costs). Identified variables, including Acute Illness Severity, Charlson Co-Morbidity and Chronic Disabling Disease that proved predictive univariately were entered into a multivariable logistic regression model to predict the bivariate of 30 days in-hospital survival. A zero truncated Poisson regression model assessed LOS and episode costs and the incidence rate ratios were calculated. Hyponatraemia was present in 22.7% of episodes and 20.3% of patients. The 30 days in-hospital mortality rate for hyponatraemic patients was higher (15.9% *vs*. 6.9% *p* < 0.001) and the LOS longer (6.3 (95% CI 2.9, 12.2) *vs.* 4.0 (95% CI 1.5, 8.2) *p* < 0.001). Both parameters worsened with the severity of the initial sodium level. Hospital costs increased non-linearly with the severity of initial hyponatraemia. Hyponatraemia remained an independent predictor of 30 days in-hospital mortality, length of stay and costs in the multi-variable model.

## 1. Introduction

Many factors influence mortality in medical patients admitted to hospital as emergencies including age, altered mental status and the presence of co-morbidities such as ischaemic heart disease, diabetes mellitus, and stroke [1]. Clinicians have sought to predict outcomes from single laboratory predictors, present at time of emergency presentation, such as disturbed sodium homeostasis [2,3,4,5], hypoalbuminaemia [6,7], hyperglycaemia [8,9,10,11], renal insufficiency [4,12,13] or combinations of these, as in laboratory scores [14,15]. Alternatively biochemical and haemodynamic variables have been combined to produce aggregate clinical scoring systems such as the Modified Early Warning Score (MEWS) [16], the Rapid Acute Physiology Score (RAPS) [17] or the Rapid Emergency Medicine Score (REMS) [18].

An abnormality in serum sodium is indicative of a disruption in the body’s water balance. Such disruptions can lead to serious clinical manifestations including convulsions, coma and even death [19]. Abnormality below the normal limits of the laboratory range is said to occur in about 15% of hospitalized patients [3]; however this estimate is best derived from direct biochemical estimates as clinical coding tends to greatly underestimate its prevalence [20]. Major morbidity and mortality have been demonstrated at the extremes of serum sodium in many patient groups including general internal medicine patients [21,22,23]. Hyponatraemia has been shown to be prognostic for specific groups of patients with cardiovascular disease, such as congestive heart failure [24,25] and pulmonary hypertension [26].

There is still debate as to whether outcomes are causally related to the presence and extent of hyponatraemia, or merely a proxy for mortality due to the underlying condition [27]. The objective of this paper was to evaluate our experience in one institution, with a prospectively collected database of more than 75,000 emergency medical admissions, between 2002 and 2013, on the problem of hyponatraemia and its relationship to outcomes (30 days in-hospital mortality and length of stay (LOS)) and total episode costs.

## 2. Methods

### 2.1. Background

St James’s Hospital (SJH) serves as a secondary care centre for emergency admissions for its local Dublin catchment area of 270,000 adults. All emergency medical admissions are referred to one of nine teams operating a 1:9 24 hours on-call roster. The “on-call” system is covered by a “physician of the day” with a post-call review round. Emergency medical patients are admitted from the Emergency Department to an AMAU opened in 2003, under the care of a physician certified in General Internal Medicine and a Subspecialty (Cardiology [1], Clinical Pharmacology [2], Respiratory Medicine [5], Rheumatology [3], Gastroenterology [5], Diabetes/Endocrinology [3])—the operation and outcome of which have been described elsewhere [28,29].

### 2.2. Data Collection

For audit purposes we employed an anonymous patient database assembling core information about each clinical episode from elements contained on the patient administration system, the national hospital in-patient enquiry (HIPE) scheme, the patient electronic record, the emergency room and laboratory systems. HIPE is a national database of coded discharge summaries from acute public hospitals in Ireland [30]. Ireland used the International Classification of Diseases, Ninth Revision, Clinical Modification (ICD-9-CM) for both diagnosis and procedure coding from 1990 to 2005 and ICD-10-CM since then.

Data held on the database includes the unique hospital number, admitting consultant, date of birth, gender, area of residence, principal and up to nine additional secondary diagnoses, principal and up to nine additional secondary procedures, and admission and discharge dates. Additional information cross-linked and automatically uploaded to the database includes physiological, haematological and biochemical parameters. Data was related to all emergency general medical patients admitted to SJH in the twelve years between 2002 and 2013.

Each emergency medical patient was referred to the team of the “on-call” Acute Medicine Consultant—on-take for a 24 hours period—most ~90% remained under the care of the admitting consultant for the duration of their admission. Approximately 9.9% of our patients stay >30 days with a median LOS of 54.8 days (IQR 38.8, 97.2). Consequently the LOS data represents a highly skewed distribution. Although the clinical episode is complete for the majority by day 30, some patients remain for social reasons related to the lack of long-term care facilities. We have therefore chosen a truncated end-point (at the 30-day endpoint) for analysis, to avoid these additional confounders.

We assessed the ability of known predictors—Acute Illness Severity [13,31], Charlson Co-Morbidity Index [32], Manchester Triage Category [33] and Chronic Disabling Score [34] to predicts outcomes (30 days in-hospital mortality and Length of Stay) and Episode Costs. Derangement of haemodynamic and physiological admission parameters has been utilised to derive an Acute Illness Severity Score that predicts clinical outcomes [13,31,35]. From modelling laboratory data collected at time of hospital admission we developed a predictive algorithm based on serum sodium, potassium, urea, albumin, red cell distribution width, white blood cell count and troponin level. The underlying principle is that deviation beyond the bounderies of “normal homeostasis” is an estimate of risk, although the relationship is non-linear and differs for each variable, it is possible to calculate an “aggregrate” risk score from the admission biochemistry profile [13]. Six groups were originally defined with a 30 days mortality risk increasing in an exponential fashion.

The Charlson Co-morbidity index provides an evaluation of Co-morbidity [32]. Co-morbidity is the presence of one or more additional disorders (or diseases) co-occurring with a primary disease or disorder. The Charlson Co-morbidity index predicts the ten-year mortality outcomes for patients who may have a range of Co-morbid conditions, such as heart disease, AIDS, or cancer (a total of 22 conditions). Each condition is assigned a score of 1, 2, 3, or 6, depending on the mortality; scores are then summed into three classifying groups (Groups 0, 1 and 2).

We recently described a chronic disability score, derived from counts of discharge ICD9/ICD10 codes, that strongly correlated with mortality and length of stay [34].

Triage categories, based on the Manchester Triage System [33] were Category 1 (resuscitation), Category 2 (very urgent), Category 3 (urgent), Category 4 (standard) and Category 5 (non-emergency).

We have data that permitted the cost of each clinical episode for emergency patients admitted between 2008–2012 to be calculated. The Republic of Ireland has proposed to introduce a Money Follows the Patient system, where a case based funding model with Diagnosed Related Groups (DRG’s), compares hospital costs, quality and efficiency. The calculation of costs per case is adjusted by reference to the relative cost weight of each DRG. The hospital costing of the price of an episode of care encompasses all costs appropriately associated with the delivery of that care including pay costs, non-pay costs and costs of diagnostics, medical services, theatres, laboratories, wards and overhead allocations as appropriate.

The hospital uses a number of standard accounting costing methodologies. The predominant approaches used in this exercise were Activity Based Costing and Absorption Costing [36,37]. Both methods are used in parallel to cost individual patient episodes of care by directly linking cost to patient clinical data (e.g., laboratory and radiology tests, inpatient bed days). The accuracy of the costing is greatly enhanced because the hospital has utilized a robust devolved accounting and budgetary framework since 2004. The financial data is validated by externally audited annual Financial Statements; in addition strong relationships between costing and clinical risk profile/outcomes data would suggest that the financial calculations provide a realistic view of the costs of care provision.

### 2.3. Statistical Methods

Descriptive statistics were calculated for background demographic data, including means/standard deviations (SD), medians/interquartile ranges (IQR), or percentages. Comparisons between categorical variables and mortality were made using chi-squared tests. The 30-day in-hospital survival outcome bivariate variable was assessed by fitting a logistic regression model for variables that were univariately predictive. Combining the significant predictors gave an AUROC of 0.87 (95% CI: 0.86, 0.87) to predict an in-hospital death by day 30. We used margins to estimate and interpret adjusted predictions for sub-groups, while controlling for other variables, using computations of average marginal effects [38]. Margins are statistics calculated from predictions of a previously fitted model at fixed values of some covariates and averaging or otherwise over the remaining covariates.

For the LOS count data, we employed a truncated Poisson regression model, including some categorical variables (e.g., disabling score groups) in the model as a series of indicator variables. The dependent variable LOS is a positive integer; it cannot have zero value. The data are truncated because there are no observations on individuals who stayed for zero days; the predictor variables were therefore regressed against LOS using the zero-truncated Poisson model. We used robust standard errors for the parameter estimates, as recommended by Cameron and Trivedi [39]. The Poisson regression coefficients are the log of the rate ratio: the rates at which events occur are the incidence rates. Thus with the Truncated Poisson regression model, we can interpret the coefficients in terms of incidence rate ratios (IRR).

As hospital costs typically have considerable heteroscedasticity, we examined the impact of the predictor variable (hyponatraemia grouping), using quantile regression; this method models the relationship between the hospital costs and the conditional quantiles (25%, median, 75%) of a predictor variable. Thus, traditional least-squares regression, requiring both normality and equal variance, does not perform well for these types of data [40]. Quantile regression, as a method, can be used to model the effects of covariates on the conditional quantiles of a response variable for such datasets [41]. The approach is robust, making no distributional assumption about the error term in a model. It is also robust to extreme points in the response space (outliers); confidence intervals for the estimated parameters are based on inversion of a rank test [42]. Quantile analysis concentrates on the dependent variable and its distribution; it is particularly appropriate where one might anticipate marked differences in the dependent variable at different quantiles of the predictor variable.

Adjusted odds ratios (OR) and 95% confidence intervals (CI) or incidence rate ratios (IRR) were calculated for those predictors that significantly entered the model (*p* < 0.10). Statistical significance at *p* < 0.05 was assumed throughout. Stata v.13.1 (Stata Corporation, College Station, TX, USA) statistical software was used for analysis.

## 3. Results

### Patient Demographics

A total of 66,933 episodes were recorded in 36,271 unique patients admitted as medical emergencies between 2002 and 2013. These episodes represented all emergency medical admissions, including patients admitted directly into the Intensive Care Unit (ICU) or High Dependency Unit (HDU), who had completed the clinical episode or who had suffered an in-hospital death, within 30 days of admission. The proportion of males and females was 48.9% and 51.1% respectively. The median (IQR) length of stay (LOS) was 5.1 (2.1, 9.8) days. The median (IQR) age was 62.2 (42.0, 77.1) years, with the upper 10% boundary at 84.5. The Charlson Comorbidity Score of 0, 1, or 2 was present by episode in 45.7%, 27.3% and 27.0% respectively. The major disease categories (MDC) by episode were respiratory (26.0%), cardiovascular (16.5%), neurological (16.2%), gastrointestinal (10.7%), hepatobilary (5.0%) and renal (4.4%).

The frequency of occurrence of each of the Acute Illness Severity risk groups was 4.3%, 9.4%, 13.5%, 15.3%, 15.4% and 30.6% and their respective 30 days in-hospital mortality risk was 0.3%, 0.12%, 0.63%, 1.4%, 4.7% and 23.9%. Charlson Co-morbidity frequency of groups 0, 1 and 2 was 53.7%, 23.0% and 23.3% and 30 days in-hospital mortality rates were 2.9%, 8.8% and 22.0% respectively. Between 2002 and 2013 with 66,933 episodes in patients admitted as a medical emergency, who completed the hospital episode or suffered an in-hospital death by day 30, only 14.7% of such episodes had no disabling disease code. The episode frequency of 1, 2, 3, or 4+ disabling codes (occurring in 1, 2, 3, or 4 or more different systems) was 28.0%, 27.7%, 18.4% and 11.2% respectively. Their respective 30 days in-hospital mortality rates were 1.2%, 4.0%, 7.7%, 13.3% and 25.4% respectively. The frequency of Manchester Triage Categories 1, 2 and 3+ at presentation was 2.1%, 39.8% and 58.2%, respectively, with respectively in hospital mortalities of 43.8%, 11.6% and 5.5%, respectively.

When split by the admission biochemical profile, hyponatraemia was present in 22.7% of episodes and 20.3% of patients. Patients with hyponatraemia were older (67.1 years (49.0, 79.0)* vs.* 60.5 years (40.2, 76.3): *p* < 0.001), remained in hospital longer (6.6 days (3.1, 12.0)* vs.* 4.7 days (1.9, 9.0): *p* < 0.001) were more likely to be female (51.4%* vs.* 48.2%: *p* < 0.001) (Table 1); patients who were readmitted had a much higher co-morbidity burden (Charlson Co-morbidity Index). The overall calculated 30 days mortality rate with hyponatraemia at admission was higher, whether calculated by episode (7.7% and 3.8% *p* < 0.001) or by unique patients—last admission only if >1 admission (15.9% and 6.9% *p* < 0.001). Patients with hyponatraemia was much more likely to be >60 years, have more chronic disabling disease (three or more disabling conditions) and more acute illness severity (Group 5: 55.8%* vs.* 33.4%; *p* < 0.001) (Table 2).

**Table 1:** Characteristics of Emergency Medical Admissions by admission Na^+ ^status.

Variable	Level	>Na^+^ ≥ 135 mEq/L	Hyponatraemia	*p*-Value
*N*		51,749	15,183	
Gender	Male	24,924 (48.2%)	7809 (51.4%)	<0.001
	Female	26,825 (51.8%)	7374 (48.6%)	
Outcome	Alive	49,762 (96.2%)	14,012 (92.3%)	<0.001
	Died	1987 (3.8%)	1171 (7.7%)	
Age (years: IQR)		60.5 (40.2, 76.3)	67.1 (49.0, 79.0)	<0.001
LOS (days: IQR)		4.7 (1.9, 9.0)	6.6 (3.1, 12.0)	<0.001
Manchester Triage	3	30,661 (59.2%)	8655 (57.0%)	<0.001
	2	20,253 (39.1%)	6316 (41.6%)	
	1	835 (1.6%)	212 (1.4%)	
Charlson Index	0	25,167 (48.6%)	5420 (35.7%)	<0.001
	1	13,853 (26.8%)	4396 (29.0%)	
	2	12,729 (24.6%)	5367 (35.3%)	

Manchester Triage 30 days in-hospital mortality rates were 5.5%, 11.6%, and 43.8% for Triage Groups 3+, 2 and 1 respectively; Charlson Co-morbidity groups 0, 1 and 2 had 30 days in-hospital mortality rates of 2.9%, 8.8% and 22.0% respectively; IQR: Interquartile Range; LOS: Length of Stay.

**Table 2:** Age, disabling disease and acute illness severity by admission Na^+^ status.

Variable	Level	>Na^+^ ≥ 135 mEq/L	Hyponatraemia	*p*-Value
Age Profile (years)	10–39	12,856 (24.8%)	2366 (15.6%)	<0.001
	40–59	12,687 (24.5%)	3550 (23.4%)	
	60–74	11,927 (23.1%)	3985 (26.2%)	
	85+	9864 (19.1%)	3572 (23.5%)	
Disabling Score	0	6622 (12.8%)	937 (6.2%)	<0.001
	1	13,446 (26.0%)	3067 (20.2%)	
	2	14,998 (29.0%)	4574 (30.1%)	
	3	10,234 (19.8%)	3848 (25.3%)	
	4	6449 (12.5%)	2757 (18.2%)	
Acute Illness Severity	1	1876 (4.0%)	109 (0.7%)	<0.001
	2	4306 (9.3%)	391 (2.7%)	
	3	6844 (14.7%)	990 (6.8%)	
	4	8481 (18.2%)	1984 (13.6%)	
	5	9472 (20.4%)	2949 (20.3%)	
	6	15,504 (33.4%)	8125 (55.8%)	

The frequency by patient and Chronic Disabling Score of 0, 1, 2, 3 or 4+ points was 14.7%, 28.0%, 27.7%, 18.4% and 11.2%; their respective 30-day in-hospital mortality rates were 1.2%, 4.0%, 7.7%, 13.4%, and 25.4% respectively. The frequency of Acute Illness Severity Groups 1, 2, 3, 4, 5 and 6 was 4.3%, 9.4%, 13.5%, 15.3%, 15.4% and 30.6%; their respective 30-day in-hospital mortality rates were 0.13%, 0.12%, 0.63%, 1.4%, 4.7% and 23.9% respectively.

**Figure 1:**
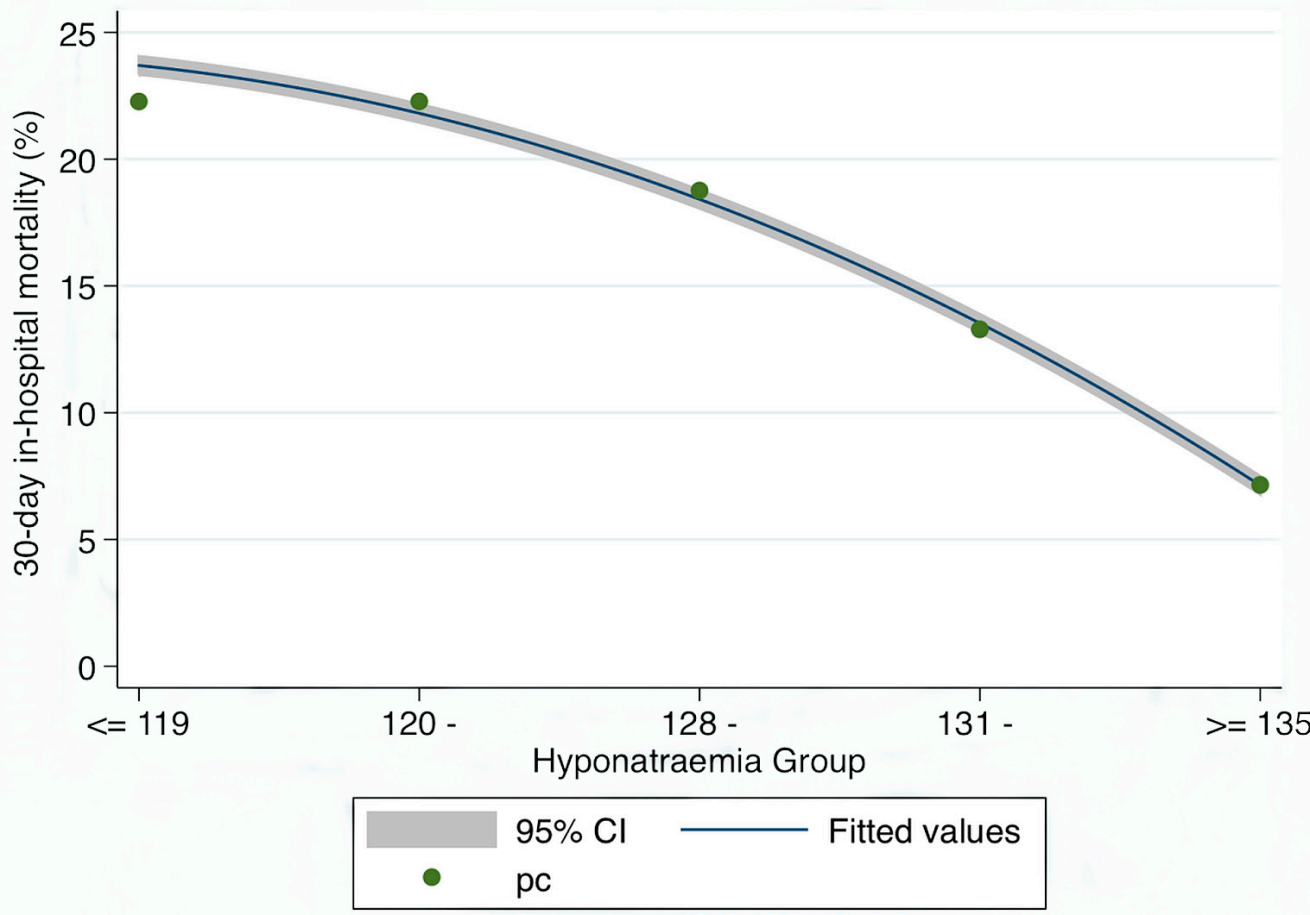
Relationship between 30 days in-hospital mortality and admission sodium level. The Incidence Rate Ratios for length of stay (LOS) (compared with the ≥135 mEq/L group) increased as the Na level fell thus: 131 mEq/L + OR 1.99 (95% CI 1.81, 2.19), 128 mEq/L ± OR 3.00 (95% CI 2.60, 3.46), 120 mEq/L ± OR 3.72 (95% CI 3.20, 4.33) and <119 mEq/L ± OR 3.72 (95% CI 2.72, 5.09).

Hyponatraemia and 30 days in-hospital Mortality (Figure 1).

Between 2002 and 2013, the overall 30 days in-hospital mortality for hyponatraemia was significantly higher, whether calculated by episode (7.7%* vs.* 3.8%: *p* < 0.001) or by unique patient (15.9%* vs.* 6.9%: *p* < 0.001). The Odds Ratio for such a death increased with the level of hyponatraemia at admission (compared with an admission Na^+^ ≥ 135 mEq/L)—for ≥131 to <135 mEq/L—OR 1.99 (95% CI 1.81, 2.19), for ≥128 to <131 mEq/L—OR 3.00 (95% CI 2.60, 3.46), for ≥120 to <128 mEq/L—OR 3.72 (95% CI 3.20, 4.33) and finally <119 mEq/L—OR 3.72 (95% CI 2.72, 5.09). The overall risk of a 30 days in-hospital death for hyponatraemia when fully adjusted for Acute Illness Severity, Age, Chronic Disabling Disease, Manchester Triage Admission Category and Charlson Co-Morbidity Score was 1.38 (95% CI 1.26, 1.51). The fully adjusted risk by hyponatraemia groups were for ≥131 to <135 mEq/L—OR 1.22 (95% CI 1.09, 1.36), for ≥128 to <131 mEq/L—OR 1.51 (95% CI 1.28, 1.77), for ≥120 to <128 mEq/L—OR 1.75 (95% CI 1.47, 2.08) and finally <119 mEq/L—OR 2.07 (95% CI 1.45, 2.94).

**Figure 2:**
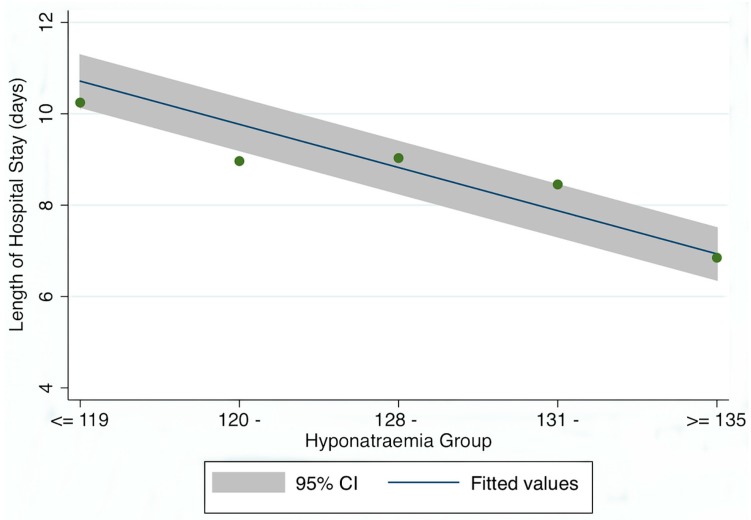
Relationship between Length of Hospital Stay (LOS) and admission sodium level. The Odds Rates of a death (compared with the ≥135 mEq/L group) increased as the Na level fell thus: 131 mEq/L + IRR 1.25 (95% CI 1.22, 1.29), 128 mEq/L ± IRR 1.36 (95% CI 1.30, 1.42), 120 mEq/L ± IRR 1.35 (95% CI 1.29, 1.42) and <119 mEq/L ± IRR 1.54 (95% CI 1.40, 1.70).

Hyponatraemia and hospital Length of Stay (Figure 2).

A truncated Poisson regression model also modelled the LOS in relation to the predictor variable—including the categorical variables (e.g., disabling score groups) in the model as a series of indicator variables. As the dependent variable LOS is truncated (observations are only positive), the different distribution requires an approach such as using the zero-truncated Poisson model [38]. The hospital Length of Stay (LOS) for hyponatraemia was significantly higher, whether calculated by episode (6.6 (95% CI 3.1, 12.0) *vs.* 4.7 (95% CI 1.9, 9.0) *p* < 0.001) or by unique patient (6.3 (95% CI 2.9, 12.2) *vs.* 4.0 (95% CI 1.5, 8.2) *p* < 0.001). The incidence rate ratios increased with the level of hyponatraemia at admission (compared with an admission Na^+^ ≥ 135 mEq/L) for ≥131 to <135 mEq/L—IRR 1.25 (95% CI 1.22, 1.29), for ≥128 to <131 mEq/L—IRR 1.36 (95% CI 1.30, 1.42), for ≥120 to <128 mEq/L—IRR 1.35 (95% CI 1.29, 1.42) and for <119 mEq/L—IRR 1.54 (95% CI 1.40, 1.70).

The overall LOS with hyponatraemia, when fully adjusted for Acute Illness Severity, Age, Chronic Disabling Disease, Manchester Triage Admission Category and Charlson Co-Morbidity Score was 1.36 (95% CI 1.33, 1.39). The fully adjusted risk by hyponatraemia groups were for ≥131 to <135 mEq/L—IRR 1.05 (95% CI 1.02, 1.08), for ≥128 to <131 mEq/L—IRR 1.05 (95% CI 1.01, 1.10), for ≥120 to <128 mEq/L—IRR 1.03 (95% CI 0.98, 1.08) and finally for <119 mEq/L—IRR 1.16 (95% CI 1.06, 1.28).

**Figure 3:**
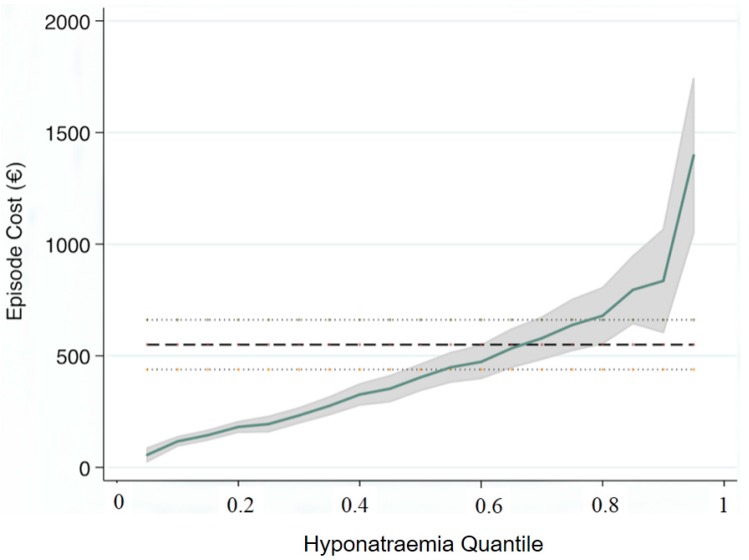
Quantile Regression (95% CI) demonstrating the change in coefficients of episode cost over the distribution of the predictor variable (Grades of admission hyponatraemia). The standard OLS regression model (hatched lines) over-estimated the costs at lower and underestimated at upper cost quantiles. Formal testing for heteroscedasticity was significant (chi^2^ (5) = 102: Prob > chi^2^ = 0.0001).

Hyponatraemia and hospital Episode Cost (Figure 3, Table 3).

**Table 3:** Quantile regression parameters of Episode Cost by Predictor Distribution.

Variables	Quantile	Parameter	95% CI
Illness Severity	0.25	43	(17, 68)
Charlson Index		131	(79, 184)
Disabling Disease		528	(490, 563)
Triage Group		−24	(−88, 41)
Hyponatraemia		194	(146, 242)
Illness Severity	0.5	153	(114, 192)
Charlson Index		260	(180, 340)
Disabling Disease		833	(777, 889)
Triage Group		−96	(−194, 3)
Hyponatraemia		403	(330, 477)
Illness Severity	0.75	434	(362, 506)
Charlson Index		559	(412, 706)
Disabling Disease		1220	(1117, 1323)
Triage Group		−21	(−202, 160)
Hyponatraemia		638	(502, 774)

Parameter estimates (95% CI) for standard Ordinary Least Squares (OLS) model were Illness Severity: 377 (318, 436), Charlson Index: 452 (331, 573); Disabling Disease: 978 (894, 1063); Triage Category: 142 (−6, 290), and Hyponatraemia: 550 (439, 661).

Hyponatraemia at time of hospital admission was a significant predictor of in-hospital costs. Quantile regression demonstrated (Figure 3) the non-linear relationship between the predictor variable (level of admission hyponatraemia) and total hospital episode cost. The standard Ordinary Least Squares (OLS) regression model—€550 (95% CI: 439, €661) over-estimated the costs at lower and underestimated at upper cost quantiles; these at Q25 point of the duration of hyponatraemia distribution were €194 (95% CI: €146, €422), at the median of €403 (95% CI: €330, €477) but increased at the Q75 point to €638 (95% CI: €502, €774). The estimates of cost increase per unit change in quantile demonstrated that hyponatraemia was only second to chronic disabling disease as a predictor variable for hospital episode costs (Table 3).

## 4. Discussion

The current study demonstrates that hyponatraemia is an independent predictor of mortality, hospital LOS, and costs in unselected general medical admissions. Our study adds to the previous literature by demonstrating in our cohort that the effect of hyponatraemia is not fully explained by illness severity or co-morbidity [27]. Furthermore our data demonstrates a biologic gradient, strengthening the support for an independent effect of hyponatraemia. Our study has shown hyponatraemia to be an extremely powerful predictor of the cost of hospital admission. Of the analysed variables only disabling disease was a more significant predictor of costs in our model.

There are significant potential clinical implications to these findings. As a minimum, the data suggest that hyponatraemia should arouse concern in clinicians that affected patients are at risk for adverse outcomes. Patients at higher risk of adverse outcomes have the potential for a greater benefit from the dedication of a physician’s limited time resources than those at low risk of events. This further justifies the role of serum sodium as a component of risk prediction scores and tools. There may be benefits to the active modification of the hyponatraemic state in these patients; however this will require further study to determine. This is of particular interest as unlike outcome predictors such as co-morbidity and disabling disease score, hyponatraemia is amenable to both preventive and corrective interventions.

The impact of serum sodium on outcomes has been evaluated in a number of previous studies. Serum sodium has been shown to be an important prognostic factor in a range of diagnostic groups including heart failure, pulmonary hypertension and subarachnoid haemorrhage [22,24,25,26]. The influence of sodium on outcomes has also been shown for intensive care unit admissions and amongst general medical admissions [23,43]. We have previously reported five year data on our patient cohort, with differential influences of acute illness severity on the strength of the association of serum sodium with mortality demonstrated for hyponatraemic and hypernatraemic patients [2]. Evaluation of the impact of hyponatraemia on outcomes by case-control methodology has shown consistent results [44,45].

Our study has several strengths. The use of a large dataset has allowed us to demonstrate that hyponatraemia is not an epiphenomenon, low serum sodium actively impacts on mortality rather than being a reflection of other risk predictors. We have included all unselected medical admissions including those sickest of the sick who are admitted to ICU or HDU. Our study therefore reflects real world clinical practice enhancing its relevance to practicing clinicians. The demonstration of a dose-response curve further enhances confidence in the veracity of the results.

Like any study ours also has limitations. We have demonstrated an association between hyponatraemia and outcomes. While we have controlled for a multitude of other risk predictors this does not neccessarily imply causation. Residual unmeasured confounders may be present which modify the effects demonstrated here. Our study was based on a retrospective cohort, we are dependant on the accurate coding and recording of data over the course of the study. In addition our study was based in a single centre, the results will require verification in other centres to establish external validity.

## 5. Conclusions

We have demonstrated an independent relationship between hyponatraemia and the key outcome measures of 30 days in-hospital mortality, length of stay, and cost of hospital admission.

## References

[1] Kellett J., Deane B. (2006). The Simple Clinical Score predicts mortality for 30 days after admission to an acute medical unit. QJM.

[2] Whelan B., Bennett K., O’Riordan D., Silke B. (2009). Serum sodium as a risk factor for in-hospital mortality in acute unselected general medical patients. QJM.

[3] Asadollahi K., Beeching N., Gill G. (2006). Hyponatraemia as a risk factor for hospital mortality. QJM.

[4] Stachon A., Segbers E., Hering S., Kempf R., Holland-Letz T., Krieg M. (2008). A laboratory-based risk score for medical intensive care patients. Clin. Chem. Lab. Med..

[5] Waikar S.S., Mount D.B., Curhan G.C. (2009). Mortality after Hospitalization with Mild, Moderate, and Severe Hyponatremia. Am. J. Med..

[6] Freire A.X., Bridges L., Umpierrez G.E., Kuhl D., Kitabchi A.E. (2005). Admission Hyperglycemia and Other Risk Factors as Predictors of Hospital Mortality in a Medical ICU Population. Chest.

[7] Goldwasser P., Feldman J. (1997). Association of serum albumin and mortality risk. J. Clin. Epidemiol..

[8] Umpierrez G.E., Isaacs S.D., Bazargan N., You X., Thaler L.M., Kitabchi A.E. (2002). Hyperglycemia: An Independent Marker of In-Hospital Mortality in Patients with Undiagnosed Diabetes. J. Clin. Endocrinol. MeTable.

[9] Suleiman M., Hammerman H., Boulos M., Kapeliovich M.R., Suleiman A., Agmon Y., Markiewicz W., Aronson D. (2005). Fasting glucose is an important independent risk factor for 30 days mortality in patients with acute myocardial infarction: A prospective study. Circulation.

[10] Stranders I., Diamant M., van Gelder R.E., Spruijt H.J., Twisk J.W., Heinem R.J., Visser F.C. (2004). Admission blood glucose level as risk indicator of death after myocardial infarction in patients with and without diabetes mellitus. Arch. Intern. Med..

[11] Krinsley J.S. (2003). Association between hyperglycemia and in-creased hospital mortality in a heterogeneous population of critically ill patients. Mayo Clin. Proc..

[12] Lim W.S., Baudouin S.V., George R.C., Hill A.T., Jamieson C., le Jeune L., Macfarlane J.T., Read R.C., Roberts H.J., Levy M.L. (2009). BTS Guidelines for the Management of Community Acquired Pneumonia in Adults. Thorax.

[13] Silke B., Kellett J., Rooney T., Bennett K., O’Riordan D. (2010). An improved medical admissions risk system using multivariable fractional polynomial logistic regression modelling. QJM.

[14] Asadollahi K., Hastings I.M., Beeching N.J., Gill G.V. (2007). Laboratory risk factors for hospital mortality in acutely admitted patients. QJM.

[15] Froom P., Shimoni Z. (2006). Prediction of hospital mortality rates by admission laboratory tests. Clin. Chem..

[16] Subbe C.P., Kruger M., Rutherford P., Gemmel L. (2001). Validation of a modified early warning score in medical admissions. QJM.

[17] Rhee K., Fisher C., Willitis N. (1987). The Rapid Acute Physiology Score. Am. J. Emerg. Med..

[18] Goodacre S., Turner T., Nicholl J. (2006). Prediction of mortality among emergency medical admissions. Emerg. Med. J..

[19] Reynolds R.M., Padfield P.L., Seckl J.R. (2006). Disorders of sodium balance. BMJ.

[20] Holland-Bill L., Christiansen C.F., Ulrichsen S.P., Ring T., Jørgensen J.O., Sørensen H.T. (2014). Validity of the International Classification of Diseases, 10th revision discharge diagnosis codes for hyponatraemia in the Danish National Registry of Patients. BMJ Open.

[21] Nardi R., Fiorino S., Borioni D., Agostini D., D’Anastasio C., Marchetti C., Muratori M. (2007). Comprehensive complexity assessment as a key tool for the prediction of in-hospital mortality in heart failure of aged patients admitted to internal medicine wards. Arch. Gerontol. Geriatr..

[22] Wartenberg K.E., Schmidt J.M., Claassen J., Temes R.E., Frontera J.A., Ostapkovich N., Parra A., Connolly E.S., Mayer S.A. (2006). Impact of medical complications on outcome after subarachnoid hemorrhage. Crit Care Med..

[23] Kraft M.D., Btaiche I.F., Sacks G.S., Kudsk K.A. (2005). Treatment of electrolyte disorders in adult patients in the intensive care unit. Am. J. Health Syst. Pharm..

[24] Gheorghiade M., Abraham W.T., Albert N.M., Gattis Stough W., Greenberg B.H., O’Connor C.M., She L., Yancy C.W., Young J., Fonarow G.C. (2007). Relationship between admission serum sodium concentration and clinical outcomes in patients hospitalized for heart failure: An analysis from the OPTIMIZE-HF registry. Eur. Heart J..

[25] Mohammed A.A., van Kimmenade R.R., Richards M., Bayes-Genis A., Pinto Y., Moore S.A., Januzzi J.L. (2010). Hyponatremia, natriuretic peptides, and outcomes in acutely decompensated heart failure: Results from the International Collaborative of NT-proBNP Study. Circ. Heart Fail..

[26] Forfia P.R., Mathai S.C., Fisher M.R., Housten-Harris T., Hemnes A.R., Champion H.C., Girgis R.E., Hassoun P.M. (2008). Hyponatremia Predicts Right Heart Failure and Poor Survival in Pulmonary Arterial Hypertension. Am. J. Respir. Crit Care Med..

[27] Chawla A., Sterns R.H., Nigwekar S.U., Cappuccio J.D. (2011). Mortality and serum sodium: Do patients die from or with hyponatremia?. Clin. J. Am. Soc. Nephrol..

[28] Rooney T., Moloney E.D., Bennett K., O’Riordan D., Silke B. (2008). Impact of an acute medical admission unit on hospital mortality: A 5 year prospective study. QJM.

[29] Conway R., O’Riordan D., Silke B. (2014). Long-term outcome of an AMAU—A decade’s experience. QJM.

[30] O’Callaghan A., Colgan M.P., McGuigan C., Smyth F., Haider N., O’Neill S., Moore D., Madhavan P. (2012). A critical evaluation of HIPE data. Ir. Med. J..

[31] O’Sullivan E., Callely E., O’Riordan D., Bennett K., Silke B. (2012). Predicting outcomes in emergency medical admissions—Role of laboratory data and co-morbidity. Acute Med..

[32] Charlson M.E., Pompei P., Ales K.L., MacKenzie C.R. (1987). A new method of classifying prognostic comorbidity in longitudinal studies: Development and validation. J. Chronic. Dis..

[33] Manchester Triage Group (2006). Emergency Triage.

[34] Chotirmall S.H., Picardo S., Lyons J., D’Alton M., O’Riordan D., Silke B. (2014). Disabling disease codes predict worse outcomes for acute medical admissions. Intern. Med. J..

[35] Mikulich O., Callaly E., Bennett K., O’Riordan D., Silke B. (2011). The increased mortality associated with a weekend emergency admission is due to increased illness severity and altered case-mix. Acute Med..

[36] Armstrong P. (2002). The Costs of Activity-Based Management. Account. Organ. Soc..

[37] Arnaboldi M., Lapsley I. (2004). Modern costing innovations and legitimation: A health care study. Abacus.

[38] Williams R. (2012). Using the margins command to estimate and interpret adjusted predictions and marginal effects. Stata J..

[39] Cameron A.C., Trivedi P.K. (2009). Microeconometrics Using Stata.

[40] Stoltzfus J., Nishijima D., Melnikow J. (2012). Why Quantile Regression Makes Good Sense for Analyzing Economic Outcomes in Medical Research. Acad. Emerg. Med..

[41] Cody C.S., Clark A.E., Thomas A.M., Cook L.J. (2012). Comparing least-squares and quantile regression approaches to analyzing median hospital charges. Acad. Emerg. Med..

[42] Koenker R., Koenker R. (1993). Confidence intervals for regression quantiles. Asymptotic Statistics, Proceedings of the Fifth Prague Symposium.

[43] Wald R., Jaber B.L., Price L.L., Upadhyay A., Madias N.E. (2010). Impact of Hospital-Associated Hyponatremia on Selected Outcomes. Arch. Intern. Med..

[44] Assen A., Abouem D., Vandergheynst F., Nguyen T., Taccone F.S., Melot C. (2014). Hyponatremia at the Emergency Department: A case-control study. Minerva Anestesiol..

[45] Tzoulis P., Bagkeris E., Bouloux P.M. (2014). A case—Control study of hyponatraemia as an independent risk factor for inpatient mortality. Clin. Endocrinol..

